# *Agistemus aimogastaensis* sp. n. (Acari, Actinedida, Stigmaeidae), a recently discovered predator of eriophyid mites *Aceria oleae* and *Oxycenus maxwelli*, in olive orchards in Argentina

**DOI:** 10.3897/zookeys.312.5520

**Published:** 2013-06-26

**Authors:** Sergio Leiva, Nestor Fernandez, Pieter Theron, Christine Rollard

**Affiliations:** 1 Fellowship, National Institute Agricultural Technology (INTA). Experimental Rural Agency, Aimogasta. 5310. La Rioja. Argentina; 2 National Council of Scientific and Technological Research (C.O.N.I.C.E.T) La Rioja University Campus. Research and Technology City. Av. Luis Mansueto de la Fuente S/N. (5300) La Rioja, Argentina; 3 Research Unit for Environmental Sciences and Management, North-West University, Potchefstroom Campus, 2520, South Africa; 4 Muséum National d’Histoire Naturelle, Département Systématique et Evolution Unité OSEB, Section Arthropodes, 57 rue Cuvier. 75231, Paris, cedex 05, France

**Keywords:** *Agistemus aimogastaensis*, new species, predator, *Aceria olea*, *Oxycenus maxwelli*, Olive orchards, Argentina

## Abstract

A new species, *Agistemus aimogastaensis*, is described with the aid of optical and Scanning Electron Microscopy. This mite is an important predator of two eriophyid mites (*Aceria oleae* and *Oxycenus maxwelli*) in olive orchards (*Olea europaea*, variety Arauco) in La Rioja Province. The problems related to eriophyids in olive orchards in Argentina are highlighted and photos of the damage on leaves and fruit are included.

## Introduction

Species of the genus *Agistemus* are considered important predators on phytophagous mites, scale insects and their eggs. Recently several studies have been done on agriculturally important crop plants, such as apple, pear and citrus orchards, blackberry fruits, coconut, coffee, and fig trees, grapevines, leguminous plants, Yerba mate trees, medicinal and ornamental plants as well as vegetable crops and the stored products of these plants (Ehara 1962; Gonzalez-Rodriguez 1961; Momen 2012; Liana and Juarez 2012; Momen 2011; Al-Atawi 2011; Marchetti and Juarez 2011; Saber and Rasmy 2010; Thakur et al. 2010; Roy et al. 2006, 2008, 2009; Fadamiro et al. 2009; Kawashima et al. 2008; Jamieson et al. 2008; Matioli et al. 2007; Gotoh and Shida 2007; Matioli and De Oliveira 2007; De Gouvea et al. 2007; El-Sawi and Momen 2006; Mineiro et al. 2006; De Vis et al. 2006; Bostanian et al. 2006; Putatunda 2005; Yousuf and Chouhan 2004; Arbabi and Singh 2002; Abou-Awad et al. 2000).

Little is known about *Agistemus* species as predators of eriophyid mites in Olive orchards (Momen 2012; Abou-Awad et al. 2010) and this is the first report of this genus from olive orchards in Argentina.

The genus *Agistemus* was erected by Summers (1960) based on type species *Caligonus terminalis* Quayle 1912. Grandjean (1944) published a work on the family Stigmaeidae, and established a series of characteristics with reference to legs and palp chaetotaxy. Further contributions to the present definition of the family were made by Gonzalez-Rodriguez (1961, 1963, 1965) and Summers and Ehara (1965).

## Material and methods

All specimens were collected individually from tree surfaces (vegetative buds, leaves, inflorescences, or fruits) and preserved in 70% ethanol. Specimens studied by means of light microscopy were macerated in lactic acid and observed in the same medium, using the open-mount technique (cavity slide and cover slip) as described by Grandjean (1949) and Krantz and Walter (2009). Drawings were made using an Olympus BHC compound microscope (Rungis, France) equipped with a drawing tube. Some specimens were studied by means of a Scanning Electron Microscope (SEM). For this purpose, specimens preserved in ethanol were carefully rinsed by sucking them several times into a Pasteur pipette, and these were then transferred to buffered glutaraldehyde (2.5%) in Sörensen phosphate buffer: pH 7.4; 0.1 m for 2 hours. After postfixation for 2 hours in buffered 2% OsO_4_ solution and rinsing in buffer solution, all specimens were dehydrated in a series of graded ethanols and dried in a critical point apparatus. Specimens were mounted on Al-stubs with double-sided sticky tape and then gold coated in a sputter apparatus (Alberti and Fernandez 1988; Alberti and Fernandez 1990a, 1990b; Alberti et al. 1991; Fernandez et al. 1991; Alberti et al. 1997; Alberti et al. 2007). For a study of the genito-anal plates and genital structures, specimens were dissected and monitored during the lactic acid maceration process (in warm 70% lactic acid) before being stained with chlorazol black E, a well-known stain (Coineau 1974). Measurements taken: total length (tip of rostrum to posterior edge of notogaster) and width (widest part of notogaster) in micrometres (μm). Leg chaetotaxy studies made using standard, polarized and phase contrast microscopes. Setal formulae of the legs include the number of solenidia (in parentheses); Setal length measured with SEM.

### Morphological terminology

Morphological terms and abbreviations used are those developed by Grandjean (1944), Summers (1960) and Gonzalez-Rodriguez (1963), Kethley (1990), We add the term: longitudinally aligned tiny round-convex elevations (*r.c.e*) in reference to structures on the postocular body.

## New taxon description

### Agistemus aimogastaensis sp. n.

urn:lsid:zoobank.org:act:21DBBD18-3CF8-42B0-9F79-41FEC0BB98D2

http://species-id.net/wiki/Agistemus_aimogastaensis

#### Etymology.

The specific epithet is dedicated to the city of Aimogasta, La Rioja, Argentina, where the specimens were found.

#### Material examined.

Holotype female and 2 female paratypes, Aimogasta, Province de La Rioja, Argentina 11-NOV-2012 deposited in Instituto Nacional de Tecnologia Agropecauria (INTA), Aimogasta, La Rioja Argentina; 4 Paratype females, same date and locality as holotype deposited in Museum National d’Histoire Naturelle, Paris, France and 4 paratypes, same date and locality as holotype deposited in Geneva Natural History Museum, Switzerland. All preserved in 70% ethanol. All type specimens were collected from vegetative buds, leaves, inflorescences and fruit of *Olea europaea*, variety Arauco.

#### Diagnosis

**(adult female). Propodosomal plate**: trapezoidal; ornamented with a faintly accentuated, polyhedral reticulate pattern; eyes clearly visible, ovoid convex, smooth; post ocular body triangular, rounded extremities, with series of longitudinally aligned small round-convex elevations, joined by thread–like strands. **Metapodosomal plate** hexagonal to polyhedral; ornamented with accentuated transverse polyhedral reticulate pattern. Wide area with fine transverse integumental striae, separating propodosomal and metapodosomal plates. **Humeral** and **intercalar** plates marginally. **Setae** *g*, *ps_1_*, *ps_2_* Similarly shaped, finely barbate, sharply tipped; *ps_3_* minutely dentate, truncate *g*, *ps_1_*, *ps_2_* larger than *ps_3_* and very different in shape and appearance in optical and SEM. **Legs**: genua II, III, IVsetal formula 0-0-0; leg IV lacks solenidion. Ambulacra with two claws and empodium with three pairs of bicapitate, fan shaped Y-raylets.

This species most closely resembles *Agistemus collyerae* Gonzalez-Rodriguez 1963, principally in relation to the setation of leg IV. However *Agistemus aimogastaensis* can be easily differentiated from the latter on account of the disposition and shape of propodosomal, metapodosomal, humeral and intercalar plates; as well as the length and disposition of dorsal setae. Specific characters given by Gonzalez-Rodriguez for *Agistemus collyerae* in relation to the unusual lengths of the *ag_2_* setae (*pg_2_ sensu* Gonzalez-Rodriguez 1963) and *g* setae (*g_1_* sensu Gonzalez-Rodriguez 1963), and the equal lengths of the other setae *ag_1_, ps_1_, ps_2_* and *ps_3_* (*g_2_*, *g_3_*, *g_4_*, *pg_1_*, Fig. 8, Gonzalez-Rodriguez 1963) is very different to the situation found in *Agistemus aimogastaensis*, where these setae are equal in size and shape; but setae *ps_3_* (*g_4_ sensu* Gonzalez-Rodriguez 1963) is completely different to the other setae, both in shape and length. Finally, another important character is the post–ocular body (*pob*) and the microsculpture around this zone. The *pob* in *Agistemus aimogastaensis* is triangular with rounded extremities, and the microsculpture around this zone is smooth to fine integumental striations; in *Agistemus collyerae* the *pob* is round and the surrounding microsculpture is a thin-walled network or reticulate.

#### Description.

***Measurements***: SEM: 325 (312–351) × 160 (152–173) Light microscopy: 336 (331–339) × 168 (166–174) (n=10).

**Figure 1.**
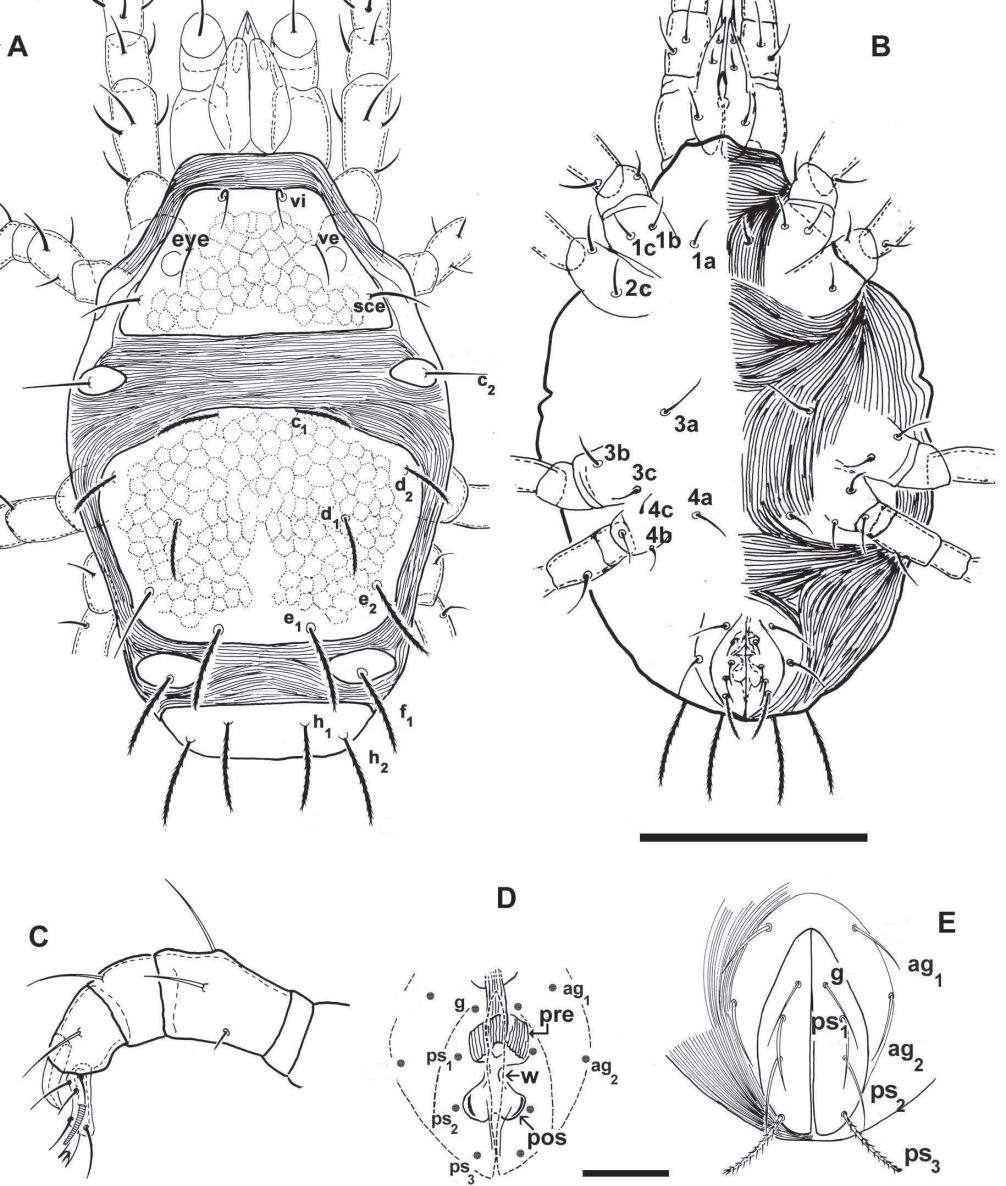
*Agistemus aimogastaensis* sp. n. Adult female, optical microscopy. **A** dorsal view **B** ventral view **C** palp **D** cuticular components of genital chamber; the anogenital covers are presented as indication of its relation to genital organs. E, anogenital covers. Abbreviations see Material and methods. Scale bars: **A**, **B**: 100 µm; **C**, **D**, **E**: 15 µm.

***Shape***: ovoid (Figures 1A, B).

***Colour***: variable. Specimens observed in reflected light: orange-yellow, slightly shiny or white. We studied specimens of different colors and all were female.

**Figure 2.**
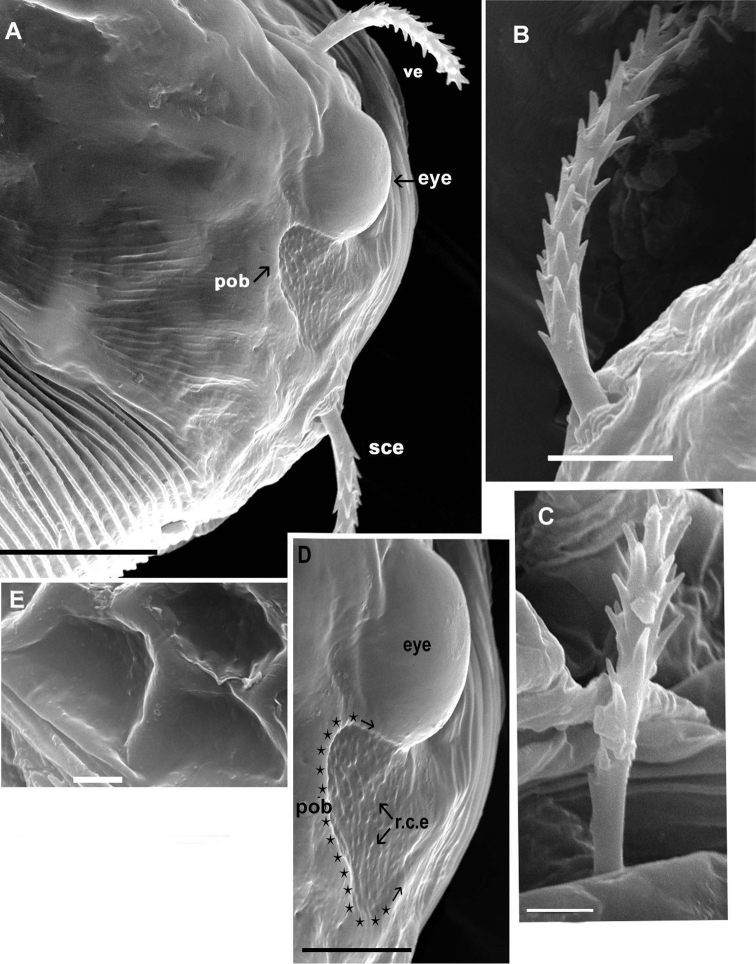
*Agistemus aimogastaensis* sp. n. Adult female, SEM. **A** eye and post ocular body, lateral view **B** external scapular setae (*sce*), lateral view **C** internal vertical setae (*vi*), lateral view **D** eye and postocular body, detail, lateral view (indicated by stars and arrow) **E** ornamentation, metapodosomal plate. Abreviations: see Material and Methods. Scale bars: **A**: 10µm; **B**, **D**, **E**: 5µm; **C**: 2µm.

***Integument***: (Figures 1A, B; 2A,D, E)

Microsculpture complicated, varying according to body region.

Propodosomal plate (P) polyhedral reticulate pattern: tiny accentuated polyhedral reticulated pattern, extending behind *vi* setal insertion and paraxially to *ve* and *sce* setal insertion, and paraxial to eye (*eye*) and post ocular body (*pob*). Near the eye and post ocular body and antiaxially to the *ve* and *sce* setal insertion smooth (Figs 2A, D). Existing paraxially to eye and *pob*, very fine integumental striae.

Metapodosomal (M) plate with polyhedral reticulate pattern, accentuate (Fig. 2D). Humeral plate (H), Intercalary plate (I), and Suranal plate (SA), more or less smooth (Fig. 1A).

Fine integumental striae covering zone between Propodosomal, Metapodosomal, Humeral, Intercalar and Suranal plates (Figs 1A, 2A).

Fine integumental striae covering venter of idiosoma, epimeral zone smooth (Fig. 1B).

Legs: cuticular surface smooth.

**Figure 3.**
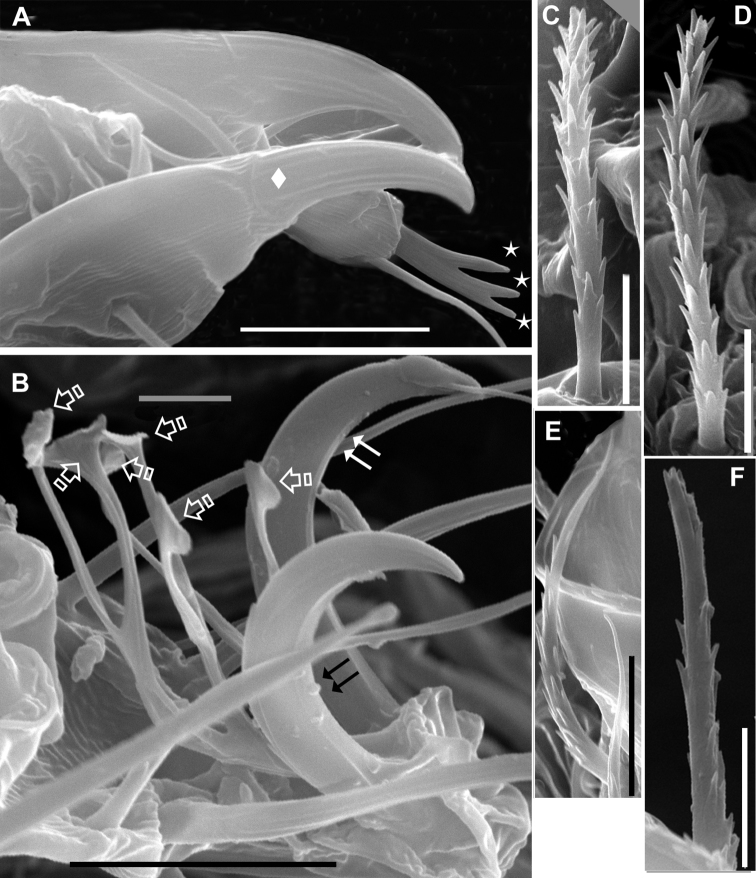
*Agistemus aimogastaensis* sp. n. Adult female, SEM. **A** palp, tibia and tarsus lateral view **B** ambulacrum leg I, lateral view. C. dorsocentral *a* setae **D** dorsolateral *la* setae **E** *ps*_2_ setae. F *ps*_3_ setae. Abreviations see material and Methods. Scale bars: **B**, **E**: 5µm; **A**, **C**, **D**, **F**: 5µm. Small stars indicate the association of eupathidia *ul*’, *ul”* and *sul*. Diamond indicates palp tibial claw. Double arrow, indicates claw, and special single arrow indicate capitate fan-shaped raylets.

***Setation*.** All dorsal setae minutely denticulate and truncate (Fig. 3C, D). Length: *vi* 12.60 (12.04-13.012); *ve* 13,78 (13.05-13.92); *sce* 18.80 (18.78-18.93); *c_2_* 20.70 (19.89-21.01; *c_1_* 19.5 (19.56-19.80); *d_1_* 16.45 (16.43-16.48); *e_1_* 18.1 (18.00-18.09); *d_2_* 19.33 (19.23-19.92); *e_2_* 17.80 (17.77-17.84); *f_1_* 17.85 (16.01-17.69); *h_1_* 14.20 (14.18-14.24); *h_2_* 17.20 (17.16-17.24).

Ventral setae: epimeric smooth (*1a*, *1b*, *1c*, *2a*, *3a*, *3b*, *3c*, *4a*, *4b*, *4c*); (*ag_1_*, *ag_2_*) and *g*, *ps_1_, ps_2_*, finely barbate (Fig. 3E), sharply tipped; *ps_3_* minutely dentate, truncate (Fig. 3F).

Length: *ag_1_* 17.61 (17.58-17.66); *ag_2_* 17.70 (17.68-17.75); *g* 17.25 (17.17-17.29); *ps_1_* 17.40 (17.38-17.43); *ps_2_* 18.20 (18.18-18.24); *ps_3_* 17.05 (17.00-17.12).

In optical microscopy the dorsal setae and genital *ps_3_* appear as dark, while epimerics, paragenital and genitals (*g*, *ps_1_*, *ps_2_*) appear transparent. Scanning Electron Micrographs depicted in Figure 3.

***Dorsal region*** (Figure 1A). Propodosomal plate (P) trapezoidal, with three pairs of setae: *vi* situated close to the anterior margin of plate; *ve* situated slightly anteriorly and paraxially to the eye and the postocular body (*pob*); *sce*, situated posteriorly and antiaxially to *pob*. All setae situated on very small protuberances.

Observation of eye and the postocular body (*pob*) (not shown on Fig. 1A) is complex, because on mites not cleared the eye and the *pob* can both be observed, but in cleared animals only the eye is visible. Position of *ve* setae complicating observation in optical microscopy. SEM permits observation of the eye in dorsal view (Fig. 2A, D) as a smooth structure, ovoid and convex in lateral view; length: 9.55 (9.48-9.56); width: 6.28 (6.26-6.29). The *pob* has a more or less triangular shape with rounded extremities (Fig. 2A, D); 5.81(5.79-5.83) in length and 5.34 (5.32-5.37) in width; a series of longitudinally aligned slightly rounded-convex elevations (*r.c.e*) present, joined by thread–like strands. In recently mounted specimens (observed in optical microscopy), the *pob* presenting small red-yellow spots, disappearing quickly; possibly these spots are the *r.c.e* observed in SEM.

Propodosomal and metapodosal plates separated by a relatively large expanse of fine integumental striae (Fig. 1A, 2A).

Humeral plate (H) ovoid, situated antiaxially to P-plate and slightly antiaxially to M-plate; setae *c_2_* insertion situated slightly paraxially to *d_2_* insertion level (Fig. 1A).

Metapodosomal plate (M) hexagonal to polyhedral.

Dorsocentral setae: insertions *c_1_* and *e_1_* situated on the same longitudinal level; *d_1_* insertion situated antiaxially to *c_1_* and *e_1_* insertion level. Dorsolateral setae: *d_2_* insertionsituated externally and close to plate margin, posteriorly to *c_1_* insertion level but anteriorly to *d_1_* insertion level; *e_2_* situated slightly paraxially to the *d_2_* insertion level and posteriorly and antiaxially to *d_1_* insertion level (Fig. 1A).

Intercalary plates (I) ovoid, situated near the body margin (Fig. 1A); *f_1_* setal insertion situated paraxially to *e_2_* insertion level and antiaxally to *e_1_* insertion level.

***Ventral region***. Epimera well defined (Fig. 1B). Setal formulae: 3-1-3-3. Anogenital region clearly discernible. Two pairs of paragenital setae: *ag_1_*, *ag_2_*; and four pairs of setae: *g*, and three anal setae *ps_1_*, *ps_2_*, *ps_3_* (see Setation). *g*, *ps_1_, ps_2_* and *ps_3_* differing in shape (See Setation).

Cuticular components of the genital chamber with *preatrium* (*pre*), saucer-shaped structure, longitudinal striate and *postatrium (post)* bilobed; between *pre* and *pos* a constriction or waist (*w*) (Fig. 1D).

**Figure 4.**
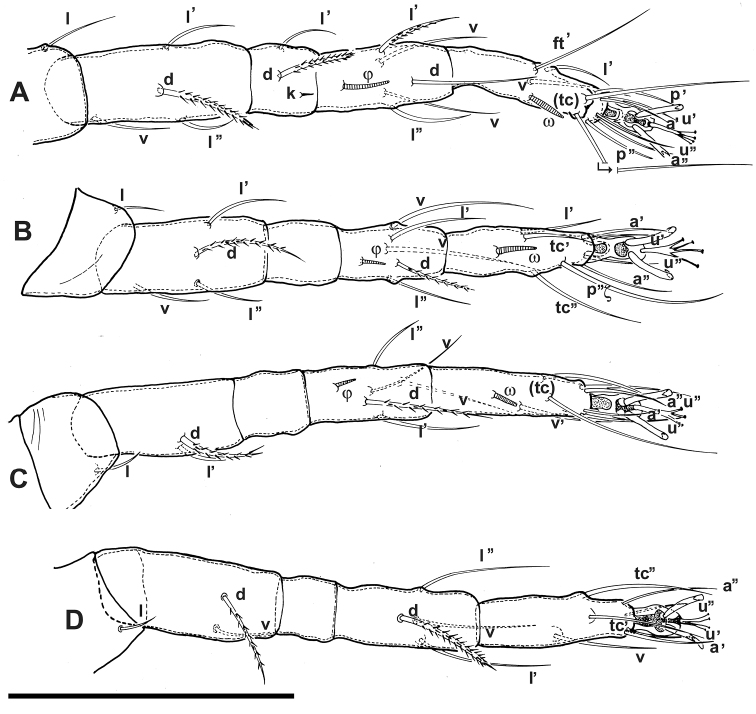
*Agistemus aimogastaensis* sp. n. Adult female, legs. All legs in dorsal view. Abbreviations: see Materials and Methods. Scale bar **A–D**: 50 µm.

***Legs*** (Figure 4A–D). All legs with ambulacrum, composed of two claws with small tooth, and an empodium with three pairs of capitate fan-shaped raylets (resembling leaves of *Ginkgo biloba* tree) (Fig. 3B).

Setal formulae (solenidia in parentheses) I (1-4-2(1)-5(1)-11(1)); setae *k* on genu I; II (1-4-0-5(1)-8(1)); III (1-2-0-5(1)-7(1)); IV (1-2-0-4-7).

Setal formulae of palp (3-1-2-8(1)) (Fig. 1C); tarsus with four eupathidia and solenidion ω; (*ul*) ζ, *sul* ζ united in fork, with typical characteristics of Stigmaeidae (Grandjean 1944). Palp tibial claw present (Fig. 3A).

#### Remarks.

The post ocular body, delimited by red-yellow spots, is clearly visible in fresh recently prepared specimens, but these spots disappear quickly making it difficult to view; this situation is similar to observations made on *Hydrozetes lemnae* (Oribatida, Hydrozetidae) and at the base of the ultrastructural studies of secondary eye(Alberti and Fernandez 1988, 1990a, b).

Our observations on cuticular components of the genital chamber using optical microscopy must be indicated as relative, and we stress that their value for taxonomic studies is limited as their main significance is only to confirm adulthood [as indicated by Summers and Ehara (1965)].

##### Problems with Olive orchards in Argentina related to eriophyid mites and their predator *Agistemus aimogastaensis* sp. n.

The Olive industry in Argentina is significant, with several provinces such as Mendoza, San Juan, San Luis, La Rioja and Catamarca producing olive fruit and their derivatives, though levels of production may vary. Olive production plays a very important socio-economic role as principal provider of employment in La Rioja and Catamarca Provinces.

In olive orchards eriophyid mites are considered a secondary pest (International Olive Council 2007; Spooner et al. 2007) relating to young trees, and a problem in greenhouses or in zones with high humidity and temperature (Spooner et al. 2007). Regrettably, in Argentina, this problem has high incidence and produces large losses in olive industry yield, reaching up to 20%.

**Figure 5.**
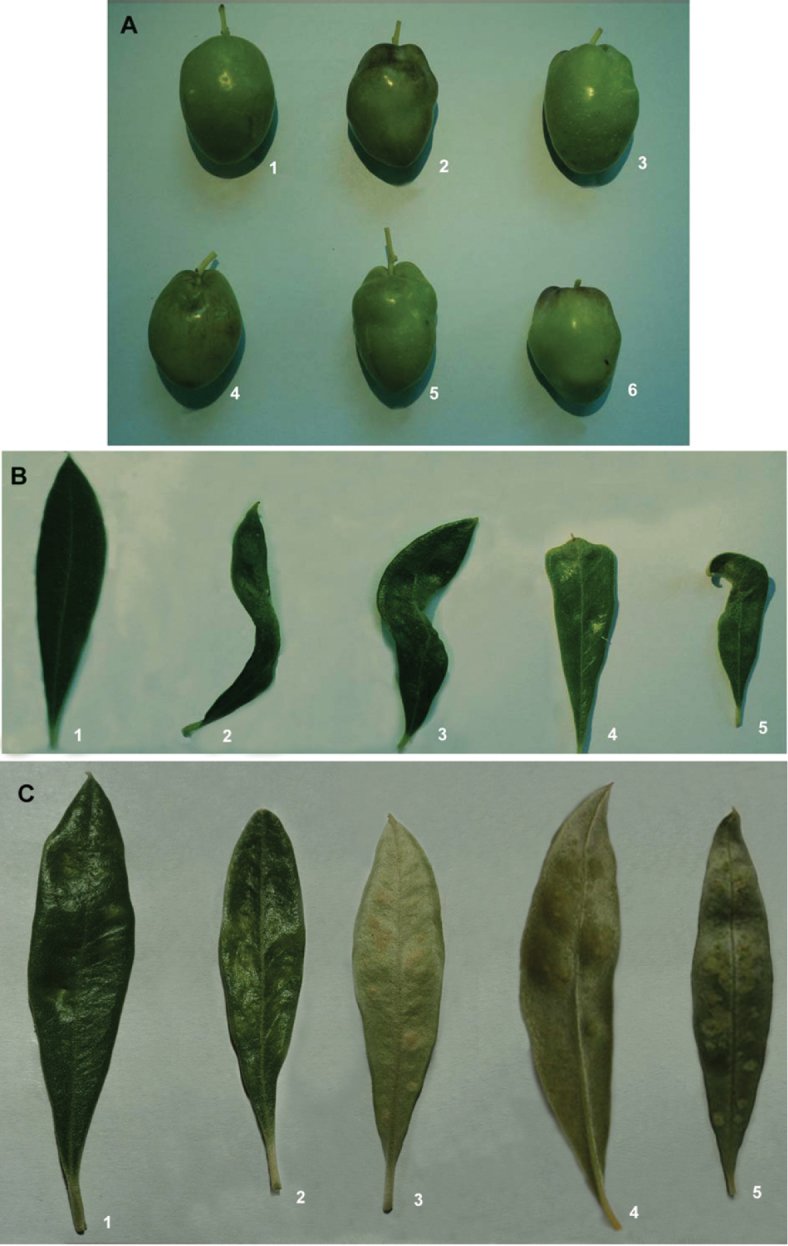
Malformations induced by eriophyid mites on leaves and fruit. **A** affected leaves **B** affected fruit. The upper left fruit is normal, others with malformations **C** young fruit attacked by *Aceria oleae* **D** detail of attack in **C.**

The predominant species of eriophyid mites found in Catamarca and La Rioja Provinces on *Olea europaea* (variety Arauco) are *Aceria oleae* and *Oxycemus maxwelli*. Of these two, *Aceria oleae* is predominant with a maximum on leaves and fruit in April and November. These two eriophyid mites cause a significant impact on regional economies due to significant fruit and leaf malformations (Figure 5).

The predator *Agistemus aimogastaensis* was found in these two provinces in large numbers, principally in relation to the population level of eriophyid mites.

The possibility exists of using this predator as biological control measure of problematic eriophyid mites. Our laboratory observations show that *Agistemus aimogastaensis* is a voracious predator, principally on *Aceria oleae*. All ontogenetic stages prey on the mites. Several studies on different predation aspects are being conducted.

## Supplementary Material

XML Treatment for Agistemus aimogastaensis
